# Behavioral Pathways Linking Hypocritical Leadership to Knowledge Hiding in Healthcare: A Three-Wave Study of Nurses

**DOI:** 10.3390/bs16101952

**Published:** 2026-10-20

**Authors:** Muhammad Asad Ullah, Mahmoud M. Abdelwahab, Muhammad Muneeb Hassan, Muhammad Ameeq

**Affiliations:** 1 Faculty of Business, Multimedia University, Jalan Ayer Keroh Lama, Melaka 75450, Malaysia; asadmpa@gmail.com (M.A.U.); ameeq7777@gmail.com (M.A.); 2 Department of Mathematics and Statistics, Faculty of Science, Imam Mohammad Ibn Saud Islamic University (IMSIU), Riyadh 11432, Saudi Arabia; mmabdelwahab@imamu.edu.sa; 3 Department of Statistics, DHQ Hospital Muzaffargarh, Muzaffargarh 34200, Pakistan; 4 Department of Statistics, GHSS Lab A/W (PECTAA), Muzaffargarh 34200, Pakistan

**Keywords:** hypocritical leadership, knowledge hiding, workplace incivility, workplace cheating behavior, ethical leadership

## Abstract

Knowledge hiding is a counterproductive workplace behavior that can undermine collaboration, organizational effectiveness, and healthcare quality. This study examined the behavioral mechanisms linking hypocritical leadership to knowledge hiding among nurses by investigating the sequential mediating roles of workplace incivility and workplace cheating behavior. Guided by social learning theory and behavioral integrity theory, a three-wave time-lagged survey was conducted among 299 registered nurses working in a tertiary healthcare hospital in Southern Punjab, Pakistan. Data were analyzed using Hayes’ PROCESS Model 6 to evaluate direct and sequential mediation effects. The findings showed that hypocritical leadership was positively associated with workplace incivility and workplace cheating behavior. The direct association between leaders’ hypocrisy and knowledge hiding was not statistically significant, whereas the indirect effects through workplace incivility and workplace cheating behavior were significant, indicating an indirect-only mediation. This suggests that the association between hypocritical leadership and knowledge hiding is established through intermediate behavioral mechanisms, while the direct effect is not statistically distinguishable from zero in this sample. Furthermore, the sequential mediation pathway from hypocritical leadership through workplace incivility and workplace cheating behavior to knowledge hiding was supported. These findings suggest that leadership hypocrisy is associated with a behavioral environment characterized by incivility and unethical workplace conduct, which in turn is related to employees’ tendency to conceal knowledge. Promoting ethical leadership and respectful workplace interactions may be associated with a lower level of counterproductive workplace behaviors and may support a collaborative knowledge-sharing culture in healthcare organizations.

## 1. Introduction

The extant literature has examined the individual and organizational level antecedents, outcomes, and underlying mechanisms of the positive leadership domains from multiple theoretical and contextual perspectives (Porkodi, 2024; Xu et al., 2025). However, there is growing criticism of the exaggerated emphasis on this so-called promising and positive organizational scholarship literature, which neglects the consequences of dysfunctional domains of leadership and management (Syed et al., 2021; Harris & Jones, 2018; Kipfelsberger & Kark, 2018; Schyns & Schilling, 2013). Empirical evidence indicates that dysfunctional leadership is associated with a range of negative behaviors within organizations, which can undermine both individual well-being and organizational performance (Ahmed et al., 2024).

Hypocritical leadership as a form of dysfunctional leadership has acquired substantial scholarly attention (Henkel & Ade, 2025). It refers to the perceived inconsistency between a leader’s espoused values and actual behaviors, such as preaching integrity while engaging in self-serving conduct (Greenbaum et al., 2012; Piccolo et al., 2010). Research suggests that hypocritical leaders act as a double-edged sword, preaching integrity, fairness, and transparency on the one hand, yet promoting self-serving behavior while engaging in unfair and deceptive practices on the other hand (Han et al., 2024). This word–deed misalignment of leaders is associated with distrust among employees, thereby leading to adverse workplace behaviors (Ni et al., 2025) such as workplace incivility (Iqbal et al., 2025; Tanskanen, 2024), workplace cheating (Han et al., 2024), and knowledge hiding behaviors (Chen et al., 2025).

Workplace incivility refers to low-intensity deviant behaviors that violate norms of mutual respect, such as rude or demeaning remarks (Cortina et al., 2001). Workplace cheating behavior encompasses acts of dishonesty aimed at gaining an unfair advantage, such as falsifying work records (Mitchell et al., 2018). Knowledge hiding refers to the intentional withholding or concealing of requested knowledge from colleagues (C. E. Connelly et al., 2012). Knowledge hiding differs from knowledge hoarding, which involves concealing knowledge that others have not explicitly requested (Yang et al., 2024). All three counterproductive workplace behaviors differ in target and form. Workplace incivility is an interpersonal violation of respect norms; workplace cheating behavior is a self-serving, rule-violating act aimed at unfair advantage; and knowledge hiding is a knowledge-specific, self-protective withholding of requested information. The proposed sequence from incivility to cheating to knowledge hiding reflects a potential behavioral escalation in which weakened respect and ethical norms may foster increasingly self-protective and instrumental responses. However, because incivility and cheating were measured concurrently, this sequence remains theoretically proposed rather than empirically established.

Systematic reviews of the nursing literature report that dysfunctional leadership-driven workplace incivility culminating among nurses ranged from 67.5% to 90.4% (Bambi et al., 2018). The literature also suggests that leaders’ hypocrisy erodes organizational culture on the one hand and impairs innovation and collaboration on the other (Schmid et al., 2018). The mechanisms linking hypocritical leadership to these outcomes are becoming clearer, though a fully integrated account remains absent. Empirical evidence suggests that leaders’ hypocrisy leads to emotional strain and reduced moral engagement among employees, which in turn is related to retaliatory workplace incivility (Ahmed et al., 2024). Furthermore, the word–deed misalignment exhibited by hypocritical leaders can normalize deceptive or manipulative employee conduct pursued for personal or professional gain (Anand et al., 2023). Such dishonest behavior aimed at gaining an unfair advantage at work is referred to as workplace cheating behavior (Mitchell et al., 2018; Farasat et al., 2022). Workplace cheating behavior, which is often associated with leaders’ hypocrisy and a workplace culture characterized by incivility, is in turn related to dysfunctional behaviors such as knowledge hiding (Farasat et al., 2020).

Despite the predictive ability of hypocritical leadership for negative work outcomes, its effect is not necessarily direct or immediate (Asadullah et al., 2022). Instead, the impact of hypocritical leadership on knowledge-hiding behaviors is channeled through specific behavioral mechanisms, such as workplace incivility and workplace cheating. Further, the mediating mechanism may involve two individual and serial paths. The separate paths may encompass workplace incivility and workplace cheating, each independently transmitting the influence of hypocritical leadership onto knowledge hiding. Empirical evidence suggests that employees experiencing leaders’ hypocrisy are more likely to engage in incivility (Agarwal et al., 2024) or cheating, which can lead to knowledge-hiding behavior and a retaliatory strategy (Adeel et al., 2023). Furthermore, a cascading pathway may explain the effect of hypocritical leadership on knowledge-hiding behaviors, where hypocritical leadership first incites incivility among employees, which in turn escalates workplace cheating behaviors that ultimately lead to heightened knowledge-hiding.

Despite the vital significance of understanding these dynamics, this question carries particular urgency in high-stakes healthcare contexts. Nurses are expected to deliver high-quality and compassionate care to patients (Siyal et al., 2021) by engaging in open communication to ensure patient safety and deliver effective care. However, these dynamics are adversely affected by dysfunctional leadership behaviors, such as hypocrisy, which in turn lead to detrimental outcomes, including knowledge hiding. Hence, it is imperative to understand the mechanisms through which hypocritical leadership is associated with incivility and cheating behavior in the healthcare sector, especially in Pakistan.

Despite these advances, the existing literature has several limitations. First, most studies examining the relationship between leadership behaviors and workplace incivility or knowledge hiding have employed cross-sectional, single-source designs (e.g., Asadullah et al., 2022; Chen et al., 2025), which limit causal inferences and may inflate relationships due to common-method bias. Second, much of this research has been conducted in Western organizational contexts, with limited attention to how cultural norms in collectivist, high-power-distance settings such as Pakistan may shape employees’ responses to hypocritical leadership. Third, few studies have examined the sequential mechanisms through which leadership hypocrisy translates into knowledge hiding, particularly in high-stakes healthcare environments where knowledge sharing is critical for patient safety. These gaps highlight the need for multi-wave, longitudinal research in non-Western healthcare settings to better understand the temporal dynamics and boundary conditions of these relationships.

This study motivates three important gaps. First, although previous research has linked hypocritical leadership with adverse employee outcomes, less is known about the behavioral mechanisms through which it is associated with knowledge hiding. Second, existing research has not sufficiently examined whether workplace incivility and workplace cheating operate as separate and sequential mediators of this relationship. Third, many prior studies have used cross-sectional, single-source designs (Asadullah et al., 2022), limiting temporal separation among the predictor, mediators, and outcome. The present study addresses these gaps by examining this model among nurses using a three-wave time-lagged design.

The current study addresses these gaps by drawing on social learning theory and behavioral integrity theory to build an integrative theoretical account of how hypocritical leadership shapes knowledge hiding through both direct and sequentially mediated pathways. SLT explains how employees learn behavioral norms by observing supervisors’ conduct, while Behavioral Integrity Theory explains how perceived word–deed inconsistency damages trust and moral credibility. Together, these perspectives suggest that hypocritical leadership may weaken respect norms, encourage self-protective and retaliatory responses, and normalize workplace incivility and cheating, which in turn may increase knowledge hiding. The individual-level associations in the proposed model have been explained through social learning theory and behavioral integrity theory. However, the attraction–selection–attrition framework provides an organizational-level context for understanding how behavioral norms may become embedded and sustained within a workplace.

This study makes three contributions to the literature on hypocritical leadership and knowledge hiding. First, although prior research has examined the relationship between hypocritical leadership and knowledge hiding among nurses, less is known about the specific behavioral mechanisms through which this association occurs. The present study extends this work by examining workplace incivility and workplace cheating behavior as distinct behavioral mechanisms that may transmit the influence of hypocritical leadership onto knowledge hiding. Second, the study integrates BIT and SLT to explain how leaders’ word–deed inconsistency may undermine moral credibility, weaken respect norms, and be associated with self-protective and deviant behaviors. Third, the study contributes empirical evidence from a three-wave, time-lagged investigation of nurses in a healthcare setting, a context in which knowledge hiding may affect not only organizational cooperation but also the quality and safety of patient care. Importantly, the novelty of this study lies not in establishing the existence of a relationship between hypocritical leadership and knowledge hiding, but in examining workplace incivility and workplace cheating behavior as separate and proposed sequential mediators using temporally separated measurements. At the same time, we acknowledge that workplace incivility and workplace cheating behavior were measured at the same time point (Time 2), which limits conclusions regarding their temporal ordering. The proposed serial pathway should therefore be interpreted as a theoretically grounded sequence that is statistically supported under the assumed ordering, rather than an empirically verified temporal chain. Examining the mediators sequentially is important because it identifies potential intervention points: reducing incivility may prevent the subsequent development of cheating and knowledge hiding, whereas examining only direct effects would conceal this escalation process.

The main purpose of this study is to examine the behavioral mechanisms linking hypocritical leadership to knowledge hiding among nurses in a healthcare setting. Specifically, we investigate whether workplace incivility and workplace cheating behavior mediate this relationship, both separately and sequentially. To guide this investigation, we address the following research questions:

RQ1: Is hypocritical leadership positively associated with knowledge hiding?
RQ2: Do workplace incivility and workplace cheating behavior mediate the relationship between hypocritical leadership and knowledge hiding?
RQ3: Do workplace incivility and workplace cheating behavior sequentially mediate this relationship?

By answering these questions, this study aims to advance understanding of how leadership hypocrisy translates into knowledge-hiding behaviors through intermediate behavioral pathways. Without an integrated account, it remains unclear whether hypocritical leadership is associated with knowledge hiding directly or whether its influence is transmitted through a sequence of interpersonal and self-protective behaviors.

## 2. Theory and Hypotheses Development

### 2.1. Theoretical Framework

This study primarily draws on Bandura’s Social Learning Theory (Bandura, 1977, 1986), particularly its observational-learning and role-modeling principles, to explain how hypocritical leadership is associated with employees’ counterproductive workplace behaviors. SLT proposes that individuals learn behavioral patterns by observing credible and salient role models, interpreting the consequences associated with those behaviors, and subsequently reproducing conduct that appears permitted, rewarded, or advantageous (Ahn et al., 2020; Horsburgh & Ippolito, 2018). Supervisors are particularly influential social models because they possess authority, control valued resources, and represent the organization in employees’ daily work experiences (Ogunfowora, 2014).

Hypocritical leadership is characterized by a discrepancy between a leader’s espoused values and enacted behavior (Han et al., 2024). For example, a leader may emphasize fairness, respect, honesty, or teamwork while simultaneously acting unfairly, disrespectfully, dishonestly, or in a self-serving manner. Such inconsistency provides employees with conflicting behavioral information. Although the leader’s verbal communication may prescribe ethical conduct, the leader’s observable actions may communicate that ethical standards are conditional and that personal interests can take precedence overstated principles.

From a social learning theory perspective, employees may therefore give greater weight to the leader’s observable behavior than to the leader’s verbal prescriptions. When employees observe that a leader violates the standards that the leader communicates, they may infer that such standards are symbolic rather than genuinely enforced. This inference can weaken the perceived consequences of misconduct and make behaviors such as disrespect, rule violation, and self-protective withholding appear more acceptable or useful. Social learning does not imply that all employees will imitate hypocrisy directly. Rather, employees may learn the broader behavioral rule that organizational standards are inconsistently applied and that self-protection is necessary.

Behavioral integrity theory (Simons, 2002; Simons et al., 2015) provides a complementary explanation of leaders’ hypocrisy. Behavioral integrity refers to the perceived consistency between a person’s words and actions. When employees perceive low behavioral integrity in their supervisor, they are likely to experience distrust, cynicism, and uncertainty regarding the supervisor’s intentions. They may experience distrust and uncertainty because the supervisor’s promises, values, and formal expectations appear unreliable. In the present context, perceived leader hypocrisy may signal that promises, ethical standards, and formal expectations cannot be relied upon. Employees may respond by reducing cooperation and by adopting defensive or retaliatory behaviors. Additionally, leader–follower value congruence can shape how employees interpret leadership behavior, with value alignment strengthening positive responses and incongruence intensifying negative perceptions such as hypocrisy and distrust (Caniëls et al., 2026). Thus, employees who strongly value integrity, respect, and knowledge sharing may respond more negatively when leaders’ enacted values conflict with these expectations.

The combination of social learning theory and behavioral integrity theory provides a more precise explanation of the proposed model. Social learning theory explains how employees observe supervisors’ conduct and learn what behaviors appear acceptable or advantageous. Behavioral integrity theory explains why perceived word–deed inconsistency undermines trust and moral credibility. Thus, employees may not copy a leader’s hypocrisy literally; instead, they may learn that ethical standards are inconsistently enforced and that self-protective conduct is necessary. This combination explains why hypocritical leadership may be associated with workplace incivility and cheating, which may subsequently be related to knowledge hiding.

The proposed sequence is therefore behavioral rather than compositional. Hypocritical leadership is expected to create conflicting ethical signals and reduce trust. These conditions may encourage workplace incivility. Incivility can further weaken reciprocity and cooperation, making self-serving and dishonest conduct more likely. Workplace cheating may then be associated with a more instrumental orientation toward workplace relationships, under which employees treat knowledge as a personal resource rather than a collective asset. Consequently, employees may report greater willingness to conceal requested knowledge. The ASA model provides a complementary organizational-level perspective, suggesting that organizations are shaped by the people they attract, select, and retain, which gradually influences shared values, norms, and culture (Schneider et al., 1995). In this context, perceived leader hypocrisy may create inconsistencies in organizational norms concerning respect, honesty, and cooperation, potentially reinforcing self-protective and unethical behaviors. However, as this study does not directly measure ASA processes or organizational culture, the model serves only as a contextual framework for understanding how behavioral norms may persist over time. Social learning theory and behavioral integrity theory remain the primary explanations for the individual-level relationships examined.

### 2.2. Hypocritical Leadership and Knowledge Hiding

A substantial body of literature indicates that dysfunctional leadership in the healthcare sector, characterized by ineffective communication and collaboration, can have severe consequences (Asadullah et al., 2022; Wang et al., 2020). Among dysfunctional leadership styles, hypocritical leadership is considered the most detrimental, adversely influencing employees’ workplace behavior. It is a double-edged sword characterized by saying one thing and doing another (Effron et al., 2018). Hypocritical leadership refers to the word–deed misalignment of leaders/supervisors. These leaders preach values that they do not exhibit through their actions or behavior (Henkel & Ade, 2025). Greenbaum et al. (2012) defined it in terms of “not walking the talk”. The extant literature shows that employees may exhibit unfavorable responses to leaders’ hypocrisy (Greenbaum et al., 2012), including turnover intentions (Hernaus et al., 2019), distrust, emotional exhaustion, stress, and anxiety (Asadullah et al., 2022). However, the most detrimental individual and organizational outcome of this leader’s hypocrisy may include knowledge hiding, in which employees adopt a coping or retaliatory mechanism (Syed et al., 2021). Knowledge hiding refers to the deliberate concealment or withholding of requested knowledge in an organization from peers, colleagues, and supervisors (C. E. Connelly et al., 2012). However, knowledge hiding is slightly different from knowledge hoarding because knowledge hoarding is concealing knowledge that others have not asked for (Yang et al., 2024). Recent studies examining the predictors of knowledge-hiding behavior have shown that distrust (Asadullah et al., 2022), leaders’ lack of moral integrity (C. Connelly et al., 2019), and their hypocrisy (Asadullah et al., 2022) are strong predictors of this behavior. Therefore, it is maintained that knowledge hiding is employees’ retaliatory behavior towards their supervisor’s hypocrisy (Farooq & Sultana, 2021).

The relationship between leaders’ hypocrisy and knowledge hiding can be explained through social learning theory and behavioral integrity theory. Social learning theory suggests that employees learn behavioral norms by observing salient role models, particularly supervisors whose actions signal what conduct is acceptable or advantageous. When leaders communicate ethical standards but fail to enact them, employees may infer that such standards are symbolic rather than genuinely enforced, which can weaken the perceived consequences of misconduct (Ahn et al., 2020; Horsburgh & Ippolito, 2018). Behavioral Integrity Theory further explains that perceived discrepancies between a leader’s words and actions undermine trust, moral credibility, and willingness to cooperate. In such circumstances, employees may perceive knowledge sharing as risky because knowledge can be used by others without fair reciprocity or recognition (Caniëls et al., 2026). Knowledge hiding may consequently serve as a self-protective or retaliatory response. However, because the effect of hypocritical leadership may operate through interpersonal and behavioral mechanisms rather than directly, the direct relationship should be treated cautiously. Based on these arguments, we expect a positive relationship between hypocritical leadership and knowledge hiding. The following hypothesis is proposed:

**H1.**  *A Leader’s hypocrisy is positively related to the knowledge-hiding behavior of employees*.

### 2.3. Hypocritical Leadership and Workplace Incivility

The existing literature suggests that dysfunctional leadership promotes negative behaviors in the workplace (Ahmed et al., 2024). The literature also suggests that leaders’ hypocrisy, as a form of dysfunctional supervisory behavior, is a strong predictor of workplace incivility that damages trust, collaboration, and knowledge sharing within the organization (Zhao et al., 2024). Employees respond to hypocritical leadership by exhibiting retaliatory behaviors such as workplace incivility (Lim et al., 2021). Workplace incivility refers to low-intensity dysfunctional behavior that violates respect norms and damages collegiality (Shin et al., 2021). These behaviors erode the organizational climate by signaling that unethical conduct is acceptable (Leiter et al., 2015). Employees working under leaders characterized by moral inconsistency and word–deed misalignment often experience frustration and emotional exhaustion (Ali et al., 2022), which consequently can lead to incivility and knowledge hiding (Almeida et al., 2022).

The examination of antecedents of incivility revealed that dysfunctional leadership behaviors such as abusive supervision (Chouhan, 2025) and leaders’ hypocrisy (Asadullah et al., 2022) could establish a degraded workplace environment where disrespect, belittling, abuse, undermining, and injustice become prevalent (Ali et al., 2022). This culture of a degraded workplace environment constitutes workplace incivility, which may have far-reaching consequences, such as knowledge hiding within the organization (Leiter et al., 2015). Hypocritical leadership may increase workplace incivility because employees observe a discrepancy between the respectful conduct leaders prescribe and the conduct they enact. According to Bandura’s SLT (Bandura, 1977, 1986), supervisors’ observable behavior provides employees with information about which interpersonal behaviors are tolerated or consequential. When leaders communicate respect but fail to exhibit it, employees may infer that interpersonal standards are negotiable and that disrespectful conduct carries limited consequences (Greenbaum et al., 2012). In addition, behavioral integrity theory suggests that perceived word–deed inconsistency can generate distrust and moral frustration, which may encourage retaliatory or defensive responses (Greenbaum et al., 2012; Simons, 2002). Based on these arguments, we expect a positive relationship between hypocritical leadership and workplace incivility. The following hypothesis is proposed:

**H2.**  *The leader’s hypocrisy is positively related to workplace incivility*.

### 2.4. Workplace Incivility and Knowledge Hiding

Workplace incivility, as a deviant workplace behavior, has far-reaching consequences for the organizational culture and functioning. As a low-intensity deviant behavior, it triggers negative responses, such as distrust, disengagement, and reduced altruistic intentions, which in turn lead to knowledge hiding as a reaction to mistreatment (Irum et al., 2020). Empirical evidence suggests that in organizations governed by dysfunctional leadership behavior and characterized by a lack of ethical orientation among workers, a culture of abuse, disrespect, and undermining is established, which fosters incivility (Wu et al., 2022). The literature also indicates that workplace incivility undermines employees’ sense of psychological safety and mutual respect (Wen et al., 2022). This workplace incivility erodes faith in organizational justice and reciprocity, encouraging knowledge withholding (Wu et al., 2022). In such cases, employees deliberately attempt to be non-resourceful by withholding knowledge to seek revenge on co-workers and supervisors (Agarwal et al., 2024).

The relationship between workplace incivility and knowledge hiding can be explained by behavioral integrity and social learning theories. Analyzing from a behavioral integrity angle, incivility conveys that the expectations of respect and teamwork are not uniformly observed, leading to a deterioration of trust and ethical standing.

This inconsistency reduces employees’ willingness to engage in pro-social behaviors such as knowledge sharing and encourages self-protective strategies, including knowledge hiding. From an SLT perspective, employees learn behavioral norms by observing what conduct is tolerated. In environments where incivility is common and unpunished, employees may infer that cooperative norms are weak and that self-protective or retaliatory behaviors carry limited consequences. Knowledge hiding may thus be learned and reproduced as an adaptive response to an uncivil climate. Based on these arguments, the following hypothesis is proposed:

**H3.**  *Workplace incivility is positively related to knowledge-hiding behavior*.

### 2.5. Hypocritical Leadership and Workplace Cheating Behavior

Hypocritical leadership, exhibited through word–deed misalignment, can establish a workplace climate characterized by ethical ambiguity. Leaders’ inability to “walk the talk” erodes moral standards within the organization, thereby fostering distrust, undermining trust, and increasing cynicism (Henkel & Ade, 2025). Empirical evidence suggests that leadership behaviors that prioritize outcomes over ethical conduct can lead to adverse responses, such as workplace cheating among employees (Farasat et al., 2020). WCB refers to the act of violating rules to promote self-interest (Yue et al., 2023). Leaders’ hypocrisy signals that ethical standards are selectively applied (Mo & Andiappan, 2024), thereby legitimizing unethical behavior as a coping mechanism against leaders’ word–deed misalignment (Adeel et al., 2023).

The behavioral integrity theory and Bandura’s Social Learning Theory can explain the relationship between leaders’ hypocrisy and workplace cheating. The behavioral-integrity lens suggests that leaders’ perceived word–deed misalignment undermines moral credibility and may lead employees to infer that ethical standards are flexible (Simons, 2002; Simons et al., 2015). Social learning suggests that employees observe supervisors’ conduct and attend to the consequences of rule violations. When leaders violate or inconsistently apply ethical standards without apparent consequences, employees may infer that similar self-serving conduct is acceptable or advantageous (Bandura, 1977, 1986). Over time, these observations may normalize dishonest practices and make workplace cheating appear to be a viable coping strategy. Based on these arguments, the following hypothesis is proposed:

**H4.**  *Leaders’ hypocrisy is positively related to workplace cheating behavior*.

### 2.6. Workplace Cheating Behavior and Knowledge Hiding

Workplace cheating behavior encompasses acts of dishonesty and deception aimed at seeking advantages or avoiding adverse consequences (Farasat et al., 2022). It has the potential to seriously harm organizational effectiveness and trust (Shen et al., 2025). As a dysfunctional organizational behavior, it can further promote negative individual and organizational outcomes such as knowledge-hiding behavior (Ahmad et al., 2025). Knowledge hiding refers to the intentional concealment of information requested by peers, colleagues, and supervisors (C. Connelly et al., 2019). Empirical evidence shows that both workplace cheating and knowledge-hiding behaviors stem from similar organizational cultures and leadership influences (Yue et al., 2023). Unethical behaviors in the workplace foster an unethical climate that normalizes negative workplace behavior (Cialdini et al., 2021). In such situations, engaging in one unethical act, such as workplace cheating behavior, can potentially lead employees to engage in other dysfunctional behaviors, including knowledge hiding (Zhong & Robinson, 2021). Hence, workplace cheating and knowledge hiding are rationalized by employees as a response to peer behavior.

The relationship between leaders’ hypocrisy and workplace cheating can be explained through Behavioral Integrity Theory and Social Learning Theory. From a behavioral integrity perspective, perceived inconsistency between a leader’s espoused values and enacted behavior undermines moral credibility, leading employees to infer that ethical standards are flexible and rule violations are tolerated. This erosion of trust increases willingness to engage in self-serving conduct, including cheating. From a Social Learning Theory perspective, employees learn norms by observing supervisors; when leaders violate rules without consequences, employees may infer that similar conduct is acceptable or advantageous. Over time, such observations can normalize dishonest practices and make workplace cheating appear as a viable coping strategy. Based on these arguments, the following hypothesis is proposed:

**H5.**  *Workplace cheating behavior is positively related to knowledge-hiding behavior*.

### 2.7. Workplace Incivility as a Mediator

Leaders’ hypocrisy, characterized by a word–deed misalignment and a lack of integrity (Mitchell et al., 2023), establishes an organizational culture of distrust and cynicism (Syed et al., 2021; Hernaus et al., 2019). This, in turn, deteriorates the interpersonal dynamics and results in workplace incivility, characterized by disrespect, belittling, abuse, undermining, and injustice (Ali et al., 2022). Workplace incivility, characterized by low-intensity counterproductive behavior, emerges in organizational cultures shaped by leaders’ hypocrisy (Chouhan, 2025). The literature shows that workplace incivility, incited by hypocritical leadership, hampers collegiality by undermining the social fabric of the workplace (Cialdini et al., 2021). Consequently, it contributes to a self-serving culture where knowledge hiding becomes the norm, thereby discouraging knowledge sharing (Anand et al., 2023). Hence, we may argue that hypocritical leadership influences knowledge hiding through the mediating mechanism of workplace incivility. This mediating mechanism can be understood through behavioral integrity theory and social learning theory. Hypocritical leadership undermines perceived behavioral integrity, weakening trust and moral credibility. Employees who experience or observe this inconsistency may respond with incivility as a form of frustration, retaliation, or adaptation to a climate where respect norms appear symbolic. Once incivility becomes prevalent, it further erodes psychological safety and reciprocity, making knowledge hiding more likely as a defensive or retaliatory response. From a social learning perspective, employees who observe that incivility is tolerated or unpunished may infer that cooperative norms are weak and that self-protective behaviors such as knowledge hiding are acceptable. Based on these arguments, the following hypothesis is proposed:

**H6.**  *Workplace incivility mediates the relationship between leaders’ hypocrisy and employees’ knowledge-hiding behavior*.

### 2.8. Workplace Cheating Behavior as a Mediator

Dysfunctional leadership behavior, such as leaders’ hypocrisy, promotes moral disengagement (Hernaus et al., 2019), distrust (Asadullah et al., 2022), and damages the ethical climate of organizations (Mitchell et al., 2023; Greenbaum et al., 2012). Employees hold the belief that self-interest and rule-breaking are necessary for survival and success in such environments (Mitchell et al., 2023), thereby creating a fertile ground for workplace cheating behavior. Workplace cheating behavior (WCB) refers to deliberate actions aimed at dishonesty to gain an unfair advantage at work (Farasat et al., 2022). WCB strengthens a culture of opportunism, self-protection, and distrust (Zhong & Robinson, 2021). Consequently, employees view knowledge as a personal asset to be leveraged rather than shared, thereby leading to knowledge concealment (C. Connelly et al., 2019). Hence, we may argue that hypocritical leadership influences knowledge hiding through the mediating mechanism of workplace cheating behavior. This mediating mechanism can be explained through behavioral integrity theory and social learning theory. From a behavioral integrity perspective, hypocritical leadership signals that ethical standards are inconsistently applied, reducing trust in organizational norms. Employees may therefore feel justified in engaging in self-serving conduct, such as cheating, as a coping response. Once cheating becomes common, it reinforces an instrumental orientation toward workplace relationships, under which knowledge is treated as a private resource, increasing knowledge hiding. From a social learning perspective, employees observe that leaders and peers engage in rule-violating behavior with limited consequences. This observation can normalize cheating and make other forms of deviance, including knowledge hiding, appear more acceptable and less risky. Over time, employees may learn that self-protective and opportunistic behaviors are effective strategies in such environments. Based on these arguments, the following hypothesis is proposed:

**H7.**  *Workplace cheating behavior mediates the relationship between leaders’ hypocrisy and employees’ knowledge-hiding behavior*.

### 2.9. The Sequential Mediating Roles of Workplace Incivility and Workplace Cheating Behavior

The existing literature depicts that leaders’ hypocrisy, characterized by a misalignment between words and deeds, fosters an unethical climate of abuse, undermining, and belittling, which significantly influences employees’ workplace behavior (Mitchell et al., 2023). This leaders’ hypocrisy causes moral confusion, distrust, and cynicism among employees, thereby degrading interpersonal relationships and collective norms (Hernaus et al., 2019). This leadership behavior is theoretically expected to be associated with two related deviant behaviors—workplace incivility and workplace cheating—which are modeled as mediators linking hypocritical leadership with knowledge hiding (Sekerka & Yacobian, 2019); workplace incivility is promoted (Leiter et al., 2015). We position workplace incivility before workplace cheating because incivility represents an initial breakdown in interpersonal respect and cooperation, whereas cheating reflects a more explicit form of self-serving rule violation. In this proposed behavioral escalation, repeated disrespect weakens cooperative norms and makes dishonest conduct easier to rationalize (Mitchell et al., 2023; Chui et al., 2021). Consequently, the entrenchment of workplace cheating behavior within the organization leads to a deterioration of norms of cooperation and fairness (Farasat et al., 2022), resulting in knowledge hiding as a self-protective mechanism (Zhong & Robinson, 2021).

This sequential mechanism can be understood through behavioral integrity theory and social learning theory. Hypocritical leadership undermines perceived behavioral integrity, weakening trust and moral credibility (Simons, 2002). Employees may initially respond with incivility as a reaction to perceived unfairness. As employees observe that incivility and cheating are tolerated or unpunished, they may learn that cooperative norms are weak and that self-protective misconduct is acceptable (Bandura, 1977, 1986). The proposed sequence therefore represents a theoretically grounded behavioral escalation process, although the concurrent measurement of the two mediators prevents us from establishing their temporal order empirically. Based on these arguments, the following hypothesis is proposed:

**H8.**  *Workplace incivility and workplace cheating behavior play a serial mediating role between hypocritical leadership and knowledge hiding*.

**Figure 1:**
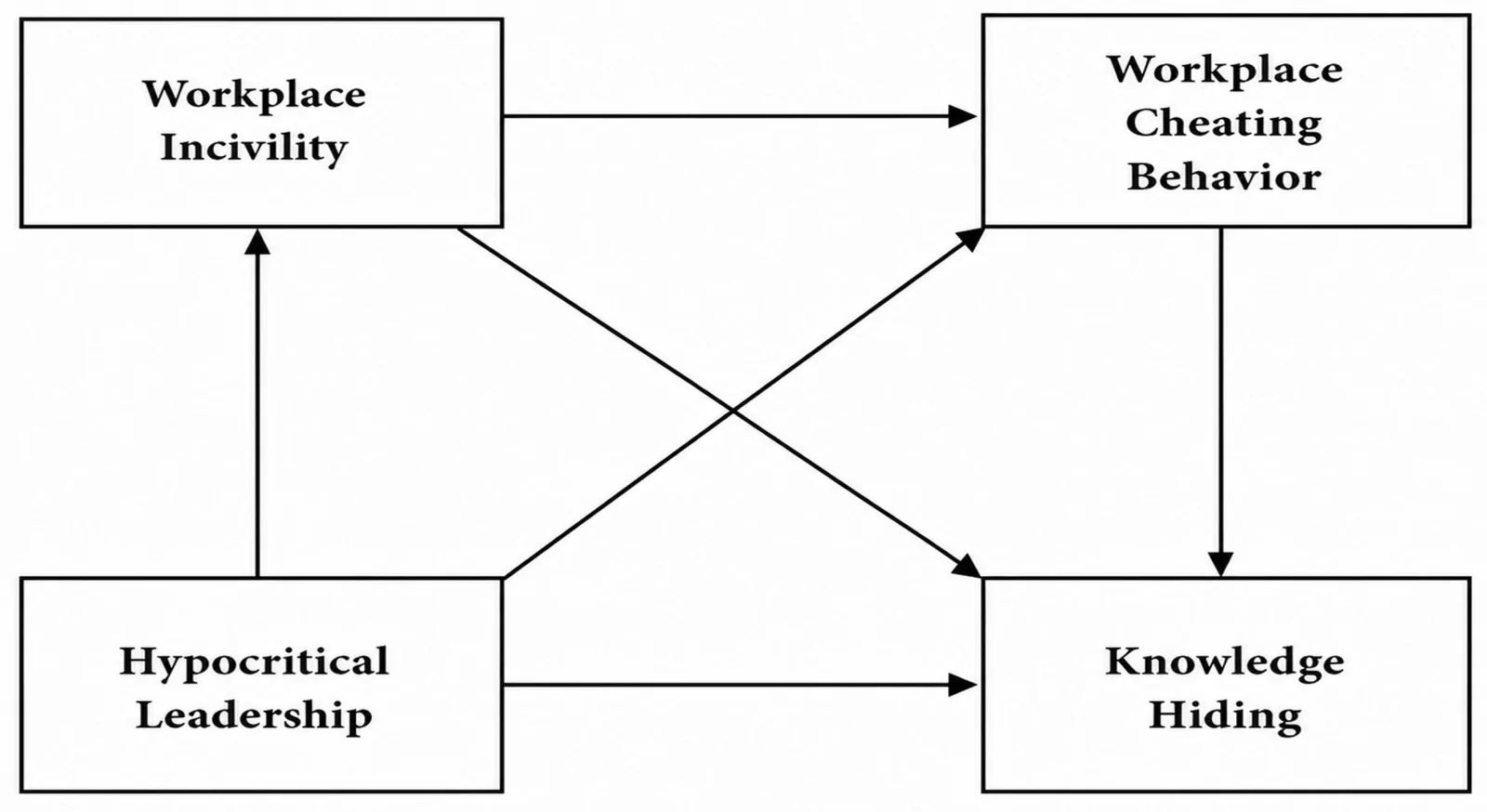
Research Model.

These arguments yield the research model shown in Figure 1, which depicts hypocritical leadership as positively associated with workplace incivility and workplace cheating behavior, and with knowledge hiding through both separate and proposed serial indirect pathways.

## 3. Material and Method

### 3.1. Research Design

We employed a three-wave time-lagged survey design to conduct this proposed study, grounded in social learning theory and behavioral integrity theory. The rationale behind selecting this design was to address issues of common method bias (CMB) and to establish temporal precedence among the study variables. Data were collected using a survey questionnaire, with responses anchored on a five-point Likert scale ranging from 1 (strongly disagree) to 5 (strongly agree).

### 3.2. Respondents and Data Collection Procedure

Respondents in this time-lagged study were nursing professionals employed at a tertiary healthcare organization in Southern Punjab, Pakistan. The total population comprised 1550 registered nurses working at the hospital and its affiliated healthcare facilities across all three shifts, as reported by the Human Resource Department. The healthcare sector was selected because of its high degree of relational interdependence and ethical sensitivity, making it an appropriate context for examining the relationships investigated in this study. Using Cochran’s formula with finite population correction (95% confidence, 5% margin of error, *p* = 0.50), the minimum required sample was approximately 309. To account for nonresponse and ensure adequate power for complex mediation, data were collected from 350 nurses. A priori power analysis indicated that 77 participants were required to detect a medium effect (f^2^ = 0.15) with three predictors, α = 0.05, and 80% power. The final sample of 299 exceeded this benchmark, supporting adequate power for the overall regression model.

A convenience sampling technique was used to select the representative sample for this study. First, a list of all the registered nurses was obtained from the HR office of the selected hospital. Later, 350 nurses who were available and willing to participate in the study were contacted during the data collection period. Participation was voluntary, and the nurses were eligible if they met the inclusion criteria. The inclusion criteria included (a) registered nurses employed at the hospital or affiliated facilities, (b) working any of the three shifts, and (c) willing to provide informed consent and complete all three survey waves. Of 350 nurses approached, 299 completed all three waves, yielding an 85% response rate. We acknowledge that convenience sampling limits the representativeness of the sample and the generalizability of the findings to other healthcare settings or professions. However, this approach is commonly used in organizational research when access to the full population is restricted and when the primary objective is to test theoretically derived relationships rather than to produce population estimates.

### 3.3. Measures and Level of Analysis

The unit of observation was the individual registered nurse who completed the questionnaire. The unit of analysis was also the individual nurse, because the proposed model examined individual-level relationships between nurses’ perceptions of hypocritical leadership and their reported workplace behaviors. The study did not aggregate responses to the ward, department, supervisor, or hospital level, and no multilevel analysis was conducted. To preserve temporal separation, hypocritical leadership and control variables were measured at Time 1, workplace incivility and workplace cheating behavior at Time 2, and knowledge hiding at Time 3.

We used all scales in their original form without modifying the wording of items or the response format. Further, we made no contextual adaptation (e.g., changing the referent from ‘supervisor’ to ‘others at work’) nor added or deleted any item. Moreover, we administered the English version of the questionnaire without translating it, as it is the medium of instruction in nursing education in Pakistan.

1. Hypocritical Leadership was measured as nurses’ perception of their supervisors’ inconsistency between espoused values and enacted behavior using a 4-item scale developed by Dineen et al. (2006). One sample item included, “I wish my supervisor would practice what he/she preaches more often.”
2. Workplace incivility was measured using a 7-item scale adopted by Cortina et al. (2001). Items are phrased from the target’s perspective (e.g., “Made demeaning or derogatory remarks about me”), capturing experienced incivility—disrespectful treatment received from others. The scale does not identify the source of workplace incivility; therefore, it cannot be attributed specifically to supervisors or hypocritical leaders. Our model proposes that leadership hypocrisy increases experienced incivility by weakening respect norms, but the data cannot distinguish its specific source.
3. Workplace Cheating Behavior was measured using a 7-item scale developed by Mitchell et al. (2018). One sample item included “Made up work activity to look better.”
4. Knowledge Hiding was measured using a 12-item scale of C. E. Connelly et al. (2012). One sample item included, “I pretended I did not know what he/she was talking about.” Knowledge hiding has been conceptualized as comprising evasive hiding, playing dumb, and rationalized hiding, but the present study models it as a single latent construct to estimate the overall association with hypocritical leadership and behavioral mediators. Because alternative multidimensional specifications were not formally compared, this measurement decision should be interpreted cautiously and examined further in future research.

### 3.4. Data Analysis and Results

Confirmatory factor analysis was conducted in AMOS to evaluate the measurement model and its fit. The direct and indirect hypotheses were then tested using Hayes’ PROCESS Macro Model 6 in SPSS Version 26. PROCESS was used to estimate the serial mediation model and generate bootstrapped confidence intervals for the indirect effects. Accordingly, the fit indices reported in the manuscript refer to the CFA measurement model, whereas the mediation coefficients and indirect effects were obtained from PROCESS Model 6. For the PROCESS analysis, mean composite scores were calculated for each construct by averaging the corresponding scale items. PROCESS therefore estimated the mediation model using observed composite scores rather than latent factor scores or a full latent-variable structural equation model. The CFA was used to evaluate the measurement properties of the constructs, whereas PROCESS was used to estimate the direct and indirect relationships among the composite variables.

### 3.5. Respondents’ Profile

Table 1 shows that 299 nurses completed the questionnaires across all three waves of the study, out of which 85 (28%) were male, while 214 (72%) were female. The results indicate that female participants dominated the sample because male nursing in Pakistan is still in its early stage of development (Younas et al., 2022). The ages of the respondents in the study ranged from 20 years to over 50 years, showing a balanced spread of the sample across all age groups. The results show that 105 (35%) nurses had 1–5 years of experience, 107 (36%) had 6–10 years of experience, and 87 (29%) had more than 10 years of experience.

### 3.6. Normality, Multicollinearity Analysis, and Common Method Variance (CMV)

Before performing inferential analysis, we conducted normality and multicollinearity analyses to examine whether the data are normally distributed and whether there are no multicollinearity issues. Results show that skewness values ranged from −0.183 (hypocritical leadership) to 1.10 (knowledge hiding), and kurtosis values ranged from −0.596 (workplace incivility) to 0.808 (knowledge hiding), indicating no substantial deviations from normality. Furthermore, the variance inflation factor (VIF) and tolerance values for all predictors also fall within the acceptable ranges of <5 and >0.2, respectively, indicating no multicollinearity concerns (Hair et al., 2010). Further, values of VIF <3.3 for all the predictors in Table 2 show that multicollinearity and CMV are within acceptable limits (Hair et al., 2010). Similarly, the value of Herman’s Single Factor Test = 31.13% <50% in Table 2 (Also annexed as Appendix A) indicates no CMV (Podsakoff et al., 2003). Further, the use of a time-lagged design also helped us address the issue of common method bias. Together with the time-lagged design, these results suggest that common-method variance is unlikely to fully account for the observed relationships, although it cannot be completely ruled out.

### 3.7. Confirmatory Factor Analysis (CFA)

Confirmatory factor analysis was conducted in AMOS to assess the measurement model. Standardized factor loadings for all the items are reported in Table 3 and ranged from 0.60 to 0.76 (*p* < 0.001) across hypocritical leadership, workplace incivility, workplace cheating behavior, and knowledge hiding. Further, composite reliability values ranged from 0.85 to 0.91, and AVE values ranged from 0.51 to 0.58. These results provide evidence of internal consistency and convergent validity for the four constructs.

### 3.8. Measurement Model Fit

**Figure 2:**
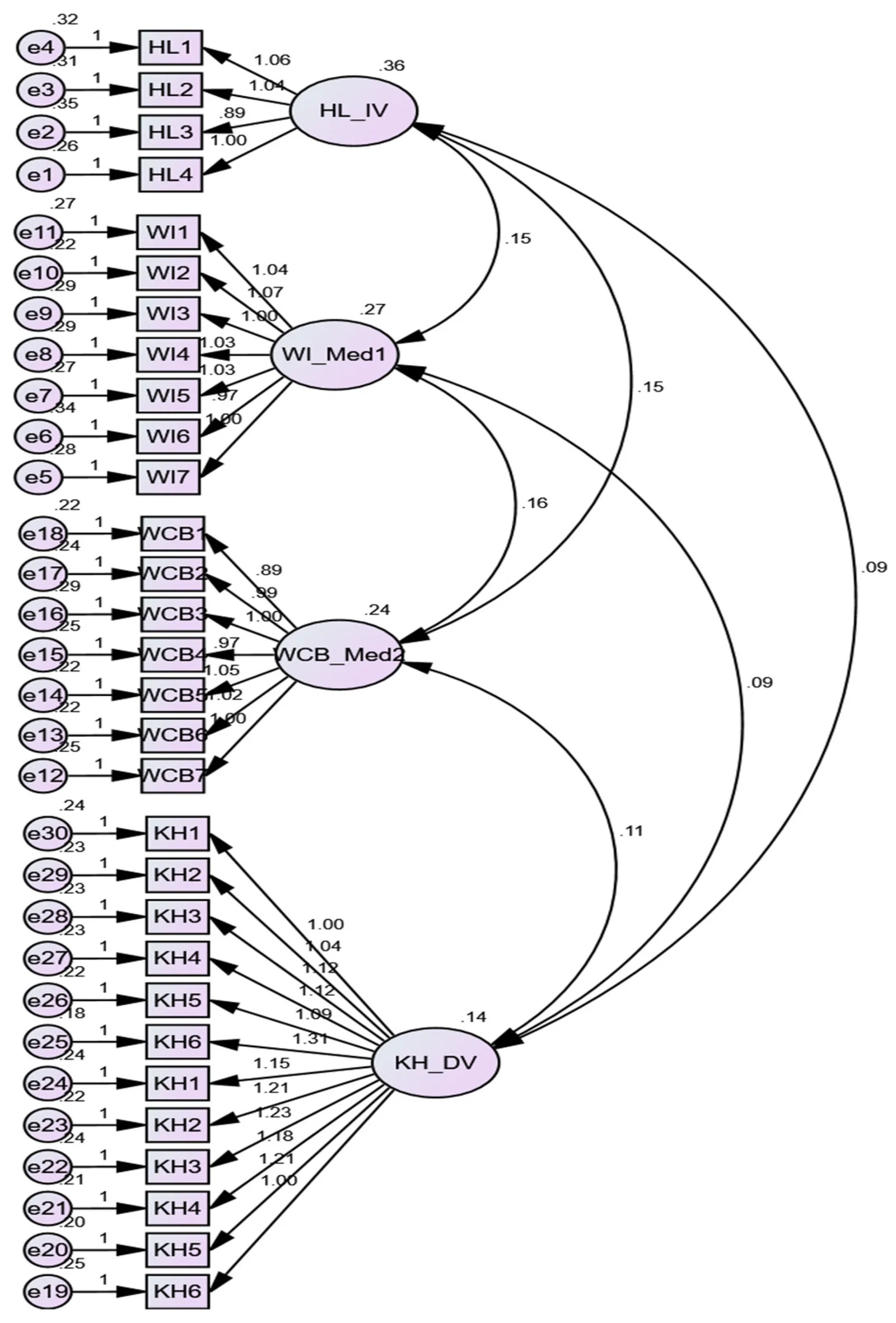
CFA for the entire Model through AMOS.

The measurement model, as shown Figure 2, was evaluated using CFA in AMOS. The fit indices at Table 4 indicated an acceptable measurement-model fit: χ2(348) = 489.15\χ2(348) = 489.15, χ2(348) = 489.15, *p* < 0.001, CMIN/df = 1.226, TLI = 0.91, CFI = 0.97, GFI = 0.91, and RMSEA = 0.03, confirming that the model accurately reflects the observed data.

### 3.9. Dimensionality of Knowledge Hiding

We conducted an EFA using principal axis factoring to examine the dimensionality of knowledge hiding. Table 5 shows high sampling adequacy (KMO = 0.943) and a significant Bartlett’s test (χ^2^(66) = 1511.86, *p <* 0.001). A one-factor solution emerged, explaining 49.48% of the variance, with all 12 items loading from 0.609 to 0.750 and the second eigenvalue = 0.841. Further, we also compared a one-factor model with a three-factor CFA model comprising evasive hiding, playing dumb, and rationalized hiding. Both the models showed acceptable fit: one-factor, χ^2^(54) = 99.62, CFI = 0.97, TLI = 0.96, RMSEA = 0.05; three-factor, χ^2^(51) = 89.95, CFI = 0.97, TLI = 0.97, RMSEA = 0.05. Although the three-factor model fit statistically better, Δχ^2^(3) = 9.67, *p* = 0.022, the improvement was modest. Given the dominant EFA factor, adequate one-factor CFA fit, and focus on overall knowledge hiding, we retained the overall KH composite. This does not imply that its three dimensions are empirically identical.

### 3.10. Reliability, AVE, and Correlation Matrix

It is evident from Table 6 that no demographic variable had a significant correlation with the main variables of the study, suggesting that there was no need to regress these variables during the primary analysis. We further found that HL had a significant positive relationship with WI (r = 0.414 **), WCB (r = 0.420 **), and KH (r = 0.335 **). Similarly, WI had a significant positive relationship with WCB (r = 0.556 **) and KH (r = 0.414 **). Furthermore, the results also revealed a significant positive relationship between WCB and KH (r = 0.533 **). These results suggested that these variables reinforce each other. Moreover, α, CR, and AVE of all the constructs satisfied Hair et al. (2010 thresholds, confirming the reliability of all the constructs.

### 3.11. Discriminant Validity

We assessed discriminant validity using both the Fornell–Larcker criterion and the heterotrait–monotrait (HTMT) ratio. It is evident in Table 7 that the square roots of AVE for the four constructs ranged from 0.670 to 0.731 and were greater than their corresponding inter-construct correlations, supporting the Fornell–Larcker criterion. We further assessed discriminant validity using the Heterotrait–Monotrait (HTMT) ratio (Henseler et al., 2015). Following the 0.90 threshold for conceptually distinct constructs (Henseler et al., 2015; Franke & Sarstedt, 2019), all HTMT values were below the recommended cutoff (range = 0.393–0.636), supporting adequate discriminant validity.

### 3.12. Mediation Analysis

Indirect and serial effects were tested using Hayes’ PROCESS macro (Version 4.2), Model 6, with 5000 bias-corrected bootstrap samples and 95% confidence intervals. Effects were significant when the intervals excluded zero, including paths through workplace incivility, workplace cheating behavior, and their serial sequence. Table 8 presented the results of serial mediation analysis generated through PROCESS Model 6 (Hayes, 2022) that investigated the effect of HL on KH through WI and WCB (HL → WI → WCB → KH). In the first stage, five hypotheses (H1–H5) were tested by examining the direct relationships between the variables.

First, we examined the direct relationship to test our first five hypotheses (H1–H5). Table 6 shows that the direct effect of HL on KH (B = 0.0708, *p* = 0.059 >0.05, 95% CI [−0.0030, 0.1446]) was not significant. Thus, Hypothesis 1 was not supported in this sample. This result does not imply that no relationship exists; rather, it indicates that any direct association was too small to be detected with adequate precision under the current design and sample size. The results further revealed that HL significantly and positively predicted WI (B = 0.3543, *p <* 0.001, 95% CI [0.2653, 0.4433]), showing that HL strongly predicted WI, supporting our 2nd hypothesis (H2). Moreover, WI had a significant positive effect on KH (B = 0.1141, *p <* 0.018, 95% CI [0.0200, 0.2082]), supporting the 3rd hypothesis (H3) of the study. Further, HL also had a significant positive effect on WCB (B = 0.179, *p <* 0.001, 95% CI [0.1001, 0.2578]), supporting our 4thhypothesis (H4). In support of our 5th hypothesis, we found that WCB had a significant positive effect on KH (B = 0.3596, *p <* 0.001, 95% CI [0.2561, 0.4632]), suggesting that WCB fosters KH among nursing professionals.

The indirect effect of HL on KH through WI was significant (B = 0.0404, 95% CI [0.0083, 0.0727]), supporting H6. The indirect effect through WCB was also significant (B = 0.0644, 95% CI [0.0314, 0.1054]), supporting H7. Finally, the indirect pathway HL → WI → WCB → KH was significant (B = 0.0535, 95% CI [0.0306, 0.0801]), supporting H8 under the assumed ordering of mediators. However, because workplace incivility and workplace cheating behavior were measured concurrently at Time 2, this result supports the proposed serial mediation model but does not establish that workplace incivility temporally precedes workplace cheating behavior.

## 4. Discussion

The non-significant direct effect indicates insufficient evidence of a direct association between hypocritical leadership and knowledge hiding, suggesting that its influence may operate primarily through intermediate behavioral mechanisms. In support of these results, C. Connelly et al. (2019) suggested that interpersonal dynamics and leadership traits shape strategic behaviors, such as knowledge hiding. From the perspective of Behavioral Integrity Theory, we argue that nurses may adapt their behavior to fit the perceived moral climate, making knowledge hiding more of a response to broader cultural norms (e.g., incivility and cheating) than to leadership behavior alone.

The results also revealed a significant positive relationship between HL and workplace incivility. From the perspective of social learning theory, employees observe and learn from supervisors’ word–deed inconsistency, which signals that respectful conduct is expected rhetorically but not necessarily practiced or enforced. The presence of hypocrisy by leaders may be associated with norms that make disrespectful conduct appear more acceptable, which in turn is related to employees’ uncivil interactions with their peers. Our findings are similar to those of Greenbaum et al. (2012), who suggested that ethical inconsistencies in leadership degrade moral standards and increase deviant behaviors such as incivility.

The significant positive relationship between HL and WCB suggests that employees may interpret leaders’ word–deed inconsistency as evidence that ethical standards are selectively enforced. Consistent with Behavioral Integrity Theory, this erosion of moral credibility may reduce employees’ commitment to formal rules. From an SLT perspective, observing that leaders violate or inconsistently apply ethical standards may make self-serving conduct appear acceptable or advantageous. Our results are consistent with those of Mitchell et al. (2018), who reported that the likelihood of justifying dishonest actions to meet personal or organizational goals increases when leaders lack moral integrity. Hypocritical leadership is positively associated with unethical standards and a climate that encourages cheating behavior.

While examining the direct effect of the mediators on the outcome variable, our results revealed a significant positive effect of WI on KH, suggesting that employees experiencing incivility are more likely to withhold knowledge. Our findings are similar to those of Chen et al. (2025), who reported that WI erodes trust, which in turn promotes defensive employee behavior. From an SLT perspective, uncivil environments signal that cooperative norms are weak, making self-protective behaviors such as knowledge hiding appear more acceptable. Similarly, WCB significantly and directly predicted KH, suggesting that employees who cheat are also more likely to conceal their knowledge. Previous studies (Shen et al., 2025; Yue et al., 2023) support these results, suggesting that employees with deceptive tendencies may adopt knowledge hiding as another form of self-protection.

The mediation analysis results revealed that both WI and WCB significantly mediated the relationship between HL and KH. This shows that HL establishes a dysfunctional climate characterized by incivility and dishonest conduct, which in turn is associated with KH among employees. Consistent with the findings of Cialdini et al. (2021), the behavioral integrity theory suggests that unethical leadership styles contribute to interpersonal mistreatment and deviance, thereby eroding trust and encouraging secrecy. Employees perceive that ethical standards are inconsistently applied, making self-protective behaviors more likely.

Finally, the indirect pathway HL → WI → WCB → KH was statistically significant and consistent with the proposed behavioral sequence under the assumed ordering of mediators. However, because workplace incivility and workplace cheating behavior were measured concurrently at Time 2, this finding supports the proposed serial mediation model but does not establish that workplace incivility temporally precedes workplace cheating behavior. The ordering of mediators is based on theory and prior work, but our design cannot confirm that increases in incivility occur before increases in cheating (Cialdini et al., 2021). From the perspective of the SLT, HL employees observe that both incivility and cheating are tolerated or unpunished. These observations can normalize a broader pattern of deviant behavior, under which knowledge hiding is seen as another acceptable form of self-protection. The sequence from hypocritical leadership to incivility, then to cheating, and finally to knowledge hiding thus reflects a behavioral escalation process driven by weakened moral credibility and learned norms of self-interest.

The overall pattern of findings suggests that leaders’ hypocrisy is positively associated with KH through the mediating mechanisms of WI and WCB, highlighting a proposed behavioral escalation process. Workplace incivility and WCB served as significant mediators of the HL–KH association. The serial mediation pathway (HL → WI → WCB → KH) is consistent with the notion of a compounded association between unethical leadership and knowledge-related misconduct (KH). These findings align with SLT and BIT, suggesting that dysfunctional leadership is associated with a climate in which deviance and knowledge hiding are more common, potentially through weakened trust and normalized misconduct. Accordingly, the main contribution of this study lies not in demonstrating that hypocritical leadership is related to knowledge hiding, but in identifying and testing workplace incivility and workplace cheating behavior as distinct and proposed sequential behavioral mechanisms using a three-wave design, while acknowledging the limitation that the two mediators were measured concurrently.

## 5. Implications

### 5.1. Theoretical Implications

This study makes three theoretical contributions. First, it extends the knowledge-hiding literature by examining workplace incivility and workplace cheating behavior as distinct behavioral mechanisms through which hypocritical leadership may be associated with knowledge hiding, rather than focusing solely on the existence of a direct relationship. The findings show that the direct effect of hypocritical leadership on knowledge hiding was non-significant, while indirect effects through workplace incivility and cheating behavior were significant, indicating indirect-only mediation.

Second, it examines a proposed sequential pathway from workplace incivility to workplace cheating that is statistically supported under the assumed ordering, offering a plausible explanation of how leadership hypocrisy is associated with knowledge hiding. This suggests that leader word–deed misalignment can adversely affect respectful interactions, promote self-serving rule violations, and subsequently relate to knowledge hiding. However, because both mediators were measured simultaneously, this sequence remains theoretically proposed rather than empirically established.

Third, the findings contribute to social learning theory and behavioral integrity Theory by showing how both perspectives can explain the proposed mechanisms. Consistent with SLT, hypocritical leadership may signal weakened respect and ethical norms, relating to workplace incivility and cheating. Behavioral integrity theory further suggests that leader word–deed inconsistency may undermine moral credibility and trust, encouraging defensive or self-serving responses. Thus, the findings highlight the importance of examining intermediate behavioral mechanisms rather than assuming a uniform direct effect on knowledge hiding.

### 5.2. Managerial Implications

The managerial perspective of our study emphasizes that organizational development managers must recognize that leaders’ hypocrisy is associated with a cascade of harmful behaviors such as incivility and cheating, which in turn is related to knowledge hiding. Hence, there is an urgent need to devise training programs that can cultivate ethical leadership to disrupt this vicious cycle and promote open knowledge sharing. Furthermore, based on the findings of this study, it is advisable to regularly monitor the organizational climate for signs of incivility and moral disengagement. Proactive climate audits can identify emergent concerns at an early stage, and leadership interventions can be formulated to realign employee behavior with organizational values, thus preventing the normalization of dysfunctional behaviors such as knowledge hiding. Hospitals should train supervisors to align their conduct with ethical and respectful standards while establishing confidential reporting mechanisms, monitoring dishonest practices, and protecting employees who raise concerns. These measures may help disrupt the proposed pathway from hypocritical leadership through workplace incivility and cheating to knowledge hiding. However, because workplace incivility and workplace cheating behavior were measured at the same time point, this sequential mechanism should be interpreted as a theoretically proposed pathway that is statistically supported under the assumed ordering, rather than an empirically verified temporal sequence.

## 6. Conclusions

This study examined the behavioral mechanisms linking hypocritical leadership to knowledge hiding among nurses in a healthcare setting. The findings indicate that hypocritical leadership is not directly related to knowledge hiding but is associated with knowledge hiding through workplace incivility and workplace cheating behavior, both separately and via a proposed serial pathway. Because the two mediators were measured concurrently, this serial pathway should be interpreted as a theoretically proposed sequence that is statistically supported under the assumed ordering, rather than an empirically established temporal chain. These results advance understanding of how leadership hypocrisy is associated with knowledge-hiding behaviors through intermediate behavioral pathways and underscore the potential value of promoting ethical leadership and respectful workplace interactions to reduce counterproductive behaviors and support a collaborative knowledge-sharing culture in healthcare organizations.

## 7. Limitations and Future Research

Although this study offers valuable insights into understanding the proposed relationships, it has several limitations. First, the study used convenience sampling within a single tertiary healthcare organization and its affiliated facilities. Therefore, findings are not statistically representative of nurses in Pakistan and should be generalized cautiously to other healthcare settings. Second, the workplace incivility measure captures experienced incivility from unspecified sources (supervisors, coworkers, or others) and does not distinguish who enacted the uncivil behavior. Consequently, while we theorize that leaders’ hypocrisy promotes workplace incivility, our data do not allow us to isolate supervisor-directed incivility from coworker-directed incivility. Future research could use source-specific incivility measures to disentangle these effects. Third, the responses can be validated by incorporating supervisor or peer ratings, as self-report measures are biased. Fourth, one of the major limitations of this study is its overreliance on negative workplace behaviors, which function serially as mediators. Future research should investigate the buffering roles of positive leadership styles (e.g., ethical or authentic leadership) and contextual variables (ethical climate or psychological safety). Comparing public and private hospitals can help researchers better understand knowledge-hiding behavior in healthcare. Fifth, although the ASA model is used to provide organizational-level context, the present study did not measure attraction, selection, attrition, workforce homogeneity, or organizational culture. Therefore, it does not directly test the core propositions of ASA theory. Future research could use multilevel or longitudinal organizational designs to examine whether ASA processes shape the workplace norms that are associated with hypocritical leadership and knowledge hiding. Sixth, all focal constructs were measured using self-report scales from the same respondents, which may introduce common-method variance and self-report bias. Although we used a time-lagged design, ensured psychological separation of measures, guaranteed respondent anonymity, and conducted Harman’s single-factor test (which did not indicate a dominant factor), these procedures cannot fully rule out common-method effects. Consequently, the observed relationships may be partly inflated, and causal inferences remain limited. Future research could use multisource data (e.g., supervisor or coworker ratings) and objective indicators to reduce common-method concerns. Finally, although we used a three-wave time-lagged design, workplace incivility and workplace cheating were measured at the same time point (Time 2). Thus, the proposed temporal sequence from incivility to cheating cannot be directly established. The ordering is based on theory and prior work, but our design cannot confirm that increases in incivility precede increases in cheating. Future research could use more fine-grained longitudinal designs (e.g., additional waves or experience sampling) to test these temporal dynamics more rigorously.

## Figures and Tables

**Table 1:** Respondents’ Profile.

Description		Frequency	Percent
Gender	Male	85	28
	Female	214	72
	Total	299	100
Age	20–30 Years	72	24
	31–40 Years	88	30
	41–50 Years	63	21
	Above 50 Years	76	25
	Total	299	100
Experience	1–5 Years	105	35
	6–10 Years	107	36
	More than 10 Years	87	29
	Total	299	100

**Table 2:** Normality and Multicollinearity Analysis.

Variable	Skewness	Kurtosis	Tolerance	VIF
HL	−0.183	−0.049	0.776	1.288
WI	0.202	−0.596	0.652	1.535
WCB	0.659	−0.264	0.647	1.545
KH	1.100	0.808	-	-
**Factor**	**Eigenvalue**	**% of Variance**	**Cumulative %**	**Factor**
1	9.341	31.14	31.14	1

**Table 3:** Standardized Factor Loadings.

Constructs	Items	Standardized Loading
Hypocritical Leadership	HL1 ← HL	0.75
(4× items)	HL2 ← HL	0.74
	HL3 ← HL	0.66
	HL4 ← HL	0.76
Workplace Incivility	WI1 ← WI	0.71
(7× items)	WI2 ← WI	0.77
	WI3 ← WI	0.69
	WI4 ← WI	0.71
	WI5 ← WI	0.72
	WI6 ← WI	0.66
	WI7 ← WI	0.70
Workplace Cheating Behavior	WCB1 ← WCB	0.67
(7× items)	WCB2 ← WCB	0.69
	WCB3 ← WCB	0.67
	WCB4 ← WCB	0.69
	WCB5 ← WCB	0.74
	WCB6 ← WCB	0.73
	WCB7 ← WCB	0.69
Knowledge Hiding	KH1 ← KH	0.61
(12× items)	KH2 ← KH	0.63
	KH3 ← KH	0.66
	KH4 ← KH	0.66
	KH5 ← KH	0.65
	KH6 ← KH	0.76
	KH7 ← KH	0.67
	KH8 ← KH	0.70
	KH9 ← KH	0.69
	KH10 ← KH	0.69
	KH11 ← KH	0.71
	KH12 ← KH	0.60

**Table 4:** Model Fitness.

Fit Index	Threshold	Result	Explanation
Chi-Square	Small expected	489.15	Good Fit
DF	>0	348	Good Fit
CMIN/df	<3.00	1.226	Good Fit
GFI	>0.90	0.91	Good Fit
TLI	>0.90	0.97	Good Fit
CFI	>0.90	0.97	Good Fit
RMSEA	<0.05	0.03	Excellent

**Table 5:** Comparison of Alternative CFA Models of KH.

Constructs	χ2	df	CFI	TLI	RMSEA
One Factor Model	99.615	54	0.97	0.96	0.05
Correlated Three Factor Model	89.945	51	0.97	0.97	0.05

**Table 6:** Reliability, AVE, Discriminant Validity, and Correlation Matrix.

Constructs	α	CR	AVE	1	2	3	4	5	6	7
1. Gender	—	—	—	1						
2. Age	—	—	—	−0.036	1					
3. Designation	—	—	—	−0.094	0.109	1				
4. HL	0.82	0.85	0.58	0.023	0.063	−0.001	1			
5. WI	0.87	0.87	0.51	−0.009	0.021	0.025	0.414 **	1		
6. WCB	0.87	0.85	0.53	−0.028	0.046	−0.020	0.420 **	0.556 **	1	
7. KH	0.91	0.91	0.57	−0.075	0.013	−0.009	0.335 **	0.414 **	0.533 **	1

Note: **. Correlation is significant at the 0.01 level (2-tailed). Diagonal are the square roots of AVE, and off-diagonal values are Pearson correlations. *p* < 0.05; *p* < 0.01. Discriminant validity is supported because each diagonal value exceeds the correlations between that construct and the other constructs. Of particular relevance, the correlation between workplace incivility and workplace cheating was 0.556, which was lower than the square root of AVE for both constructs (0.71 and 0.73, respectively). This result supports their empirical distinctiveness despite their conceptual relationship.

**Table 7:** Reliability, AVE, Discriminant Validity, and Correlation Matrix.

HTMT Ratios					Fornell–Larcker Criterion				
Construct	HL	WI	WCB	KH	Construct	HL	WI	WCB	KH
HL	—				HL	0.731			
WI	0.494	—			WI	0.418	0.709		
WCB	0.502	0.636	—		WCB	0.423	0.554	0.697	
KH	0.393	0.463	0.597	—	KH	0.34	0.413	0.53	0.67

**Table 8:** Serial Mediation Analysis.

Pathway	Effect (B)	SE	95% CI [LLCI, ULCI]	*p*-Value	Decision
Direct Effects					
HL → KH	0.0708	0.0375	[−0.0030, 0.1446]	0.0599	Not Significant
HL → WI	0.3543	0.0452	[0.2653, 0.4433]	<0.001	Significant
HL → WCB	0.179	0.0401	[0.1001, 0.2578]	<0.001	Significant
WI → KH	0.1141	0.0478	[0.0200, 0.2082]	0.018	Significant
WCB → KH	0.3596	0.0526	[0.2561, 0.4632]	<0.001	Significant
Indirect Effects					
HL → WI → KH	0.0404	0.0167	[0.0083, 0.0727]	—	Significant
HL → WCB → KH	0.0644	0.0188	[0.0314, 0.1054]	—	Significant
HL → WI → WCB → KH	0.0535	0.0127	[0.0306, 0.0801]	—	Significant
Total Indirect Effect	0.1583	0.0252	[0.1111, 0.2097]	—	Significant
Total Effect (HL → KH)	0.2291	0.0374	[0.1555, 0.3027]	<0.001	Significant

Note: KH = knowledge hiding; WI = workplace incivility; WCB = workplace cheating behavior; HL = hypocritical leadership. Indirect effects are bias-corrected bootstrap estimates based on 5000 resamples (PROCESS Model 6). Significance is inferred when the 95% confidence interval does not include zero.

## Data Availability

The data supporting the findings of this investigation are available upon request from the first author, Muhammad Muneeb Hassan. The data are not publicly available due to restrictions on their availability, which may compromise the privacy of the research participants. The corresponding author has access to the data, models, and code that support the study’s findings.

## Appendix A. Harman’s Single-Factor Test Output

We conducted an EFA with all items from the four constructs in SPSS using principal component analysis without rotation. The unrotated solution yielded 10 factors with eigenvalues greater than 1. The first (largest) factor accounted for 31.13% of the total variance, which is below the commonly used 50% threshold for flagging serious common-method concerns.

**Table A1:** Eigenvalues and Percentage of Variance Explained for the First Five Factors (Unrotated Solution).

Factor	Initial Eigenvalues			Extraction Sums of Squared Loadings		
	Total	% of Variance	Cumulative %	Total	% of Variance	Cumulative %
1	10.018	33.395	33.395	9.341	31.138	31.138
2	3.070	10.235	43.629			
3	1.833	6.111	49.740			
4	1.726	5.753	55.493			
5	0.906	3.020	58.512			
6	0.818	2.725	61.238			
7	0.793	2.642	63.880			
8	0.723	2.411	66.292			
9	0.712	2.373	68.665			
10	0.673	2.243	70.907			
11	0.639	2.129	73.037			
12	0.613	2.042	75.078			
13	0.595	1.983	77.061			
14	0.570	1.899	78.961			
15	0.548	1.825	80.786			
16	0.496	1.655	82.440			
17	0.492	1.641	84.081			
18	0.467	1.556	85.637			
19	0.459	1.529	87.166			
20	0.431	1.437	88.603			
21	0.407	1.355	89.958			
22	0.401	1.338	91.296			
23	0.385	1.283	92.579			
24	0.373	1.242	93.820			
25	0.350	1.166	94.986			
26	0.327	1.090	96.077			
27	0.321	1.071	97.148			
28	0.307	1.025	98.173			
29	0.279	0.929	99.102			
30	0.269	0.898	100.000			
